# Response to: Is it really a duplication cyst? Hypothesizing with insufficient data

**DOI:** 10.1590/1516-3180.SPMJ.2019.0184120919

**Published:** 2019-11-07

**Authors:** Zhi-Hao Huang

**Affiliations:** I MD, MSc. Surgeon, Department of Colorectal and Anal Surgery, Wuhan University Zhongnan Hospital, Wuhan, Hubei Province, China.

Dear Editor,

I would like to provide some clarifications regarding the questions raised in the letter about our article.^1^

Enteric duplications are rare but can occur anywhere along the digestive tract from the oral cavity to the rectum.^2^^,^^3^ The letter mentions that it would be possible for the lesion to arise around 100 cm proximally to the ileocecal valve. Although the majority of enteric duplications occurs intra-abdominally and over half of them are ileal duplications,^4^^,^^5^^,^^6^^,^^7^ it cannot be denied that they may also occur at other possible distances or locations in the digestive tract.

As stated in our paper,^1^ it is difficult to diagnose enteric duplications because of the non-specificity of symptoms and presentation. However, ultrasound, computed tomography scans and magnetic resonance imaging have been useful. Ultrasound can depict the characteristic location adjacent to the bowel and the two-layered wall of enteric duplications and can show the clear blood supply between the native tissue and the lesion.

The letter states that we did not mention anything about the communication of the lesion with the native intestinal lumen, or about the blood supply for the lesion, even though both of these are important features for its characterization.

**Figure 2.**
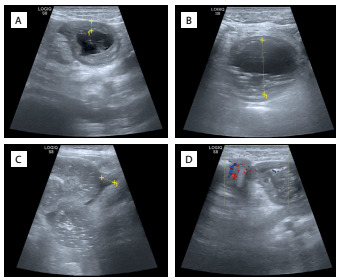
Abdominal ultrasonography: (A) A distended bowel can be seen below the umbilicus, and peristalsis is not apparent. The wall of the tube is thickened, and the lumen at the beginning of its expansion is compressed. (B) A dark area is seen between the intestines. (C) The intestinal wall is raised into the lumen, and the two intestinal tubes share the same wall of the bowel canal. (D) A small amount of colored blood flow signal can be seen on the wall of the dilated intestinal tube.

However, the requirements set forth by the author of the letter were described in Figure 2 of our paper.^1^ According to Ladd (1940), these congenital malformations involve the mesenteric side of the associated alimentary tract and share a common blood supply with the native bowel.^8^

This description conforms with the blood flow signals of Figure 2 (D) of our paper.^1^ The legend of this figure states: “A small amount of colored blood flow signal can be seen on the wall of the dilated intestinal tube”. It can also be seen in Figure 2 (D) of our paper that the native intestine and abnormal lesion share a common wall, which consists of a thick low-echo area.^1^
